# *Hemiboea siccinvolucris* (Gesneriaceae), a new species from northeastern Guangxi, China

**DOI:** 10.3897/phytokeys.274.185059

**Published:** 2026-05-12

**Authors:** Yuan Fang, Bo Pan, Peng-Wei Li, Xin-Mei Qin, Qiang Zhang, Yong-Bin Lu

**Affiliations:** 1 College of Life Sciences, Guangxi Normal University, Guilin 541006, Guangxi, China Guangxi Institute of Botany, Guangxi Zhuang Autonomous Region and the Chinese Academy of Sciences Guilin China; 2 Guangxi Key Laboratory of Plant Conservation and Restoration Ecology in Karst Terrain, Guangxi Institute of Botany, Guangxi Zhuang Autonomous Region and the Chinese Academy of Sciences, Guilin 541006, Guangxi, China Guangxi Normal University Guilin China

**Keywords:** *Hemiboea siccinvolucris*, molecular phylogeny, morphology, new species, taxonomy

## Abstract

A new species of Gesneriaceae, *Hemiboea siccinvolucris* B.Pan & Y.B.Lu, **sp. nov**., from Guangxi, China, is described. This species is morphologically similar to *H. cavaleriei*, *H. subcapitata*, and *H. liana* but can be distinguished by its densely pubescent adaxial surface and sparsely pubescent abaxial surface (vs. adaxially glabrous to sparsely pubescent and abaxially glabrous in *H. cavaleriei*; adaxially nearly glabrous to pubescent and abaxially glabrous to pubescent in *H. subcapitata*; glabrous on both surfaces in *H. liana*) and by its green abaxial surface (vs. pale green or purplish in *H. cavaleriei* and *H. subcapitata*; lilac in *H. liana*). The terminal cyme has only 1–3 flowers (vs. pseudoterminal or axillary cymes with three or more flowers in *H. cavaleriei* and *H. subcapitata*; terminal, usually 1-flowered, occasionally 2-flowered in *H. liana*). The spherical involucre dries early before anthesis (vs. spherical, with no records of early involucre desiccation in *H. cavaleriei* and *H. subcapitata*; trigonous, with no records of early involucre desiccation in *H. liana*); the stigma is reniform (vs. obtuse in *H. cavaleriei* and *H. subcapitata*; shallowly bilobed in *H. liana*). Phylogenetic analyses based on both ITS sequences and chloroplast genome data show that all sampled individuals of this taxon form a well-supported monophyletic group, supporting its recognition as a new species. Detailed morphological descriptions, line drawings, comparative diagnostic characters, and molecular phylogenetic analyses are provided.

## Introduction

*Hemiboea* C.B.Clarke is a genus of Gesneriaceae placed in subtribe Didymocarpinae, tribe Trichosporeae, subfamily Didymocarpoideae. This genus is mainly distributed in southern China, with a few species occurring in northern Vietnam, northern Laos, and Japan (Weber 2004; Li and Wang 2005). According to the most recent checklist of Chinese Gesneriaceae, *Hemiboea* exhibits a distinct diversification pattern centered on the karst regions of Southwest China. The Guangxi Zhuang Autonomous Region serves as the primary diversification hotspot with the highest species richness, while high-frequency occurrences radiate into the neighboring provinces of Guizhou and Yunnan (Tan et al. 2025). Species of *Hemiboea* are usually perennial herbs. Stems ascending, basal stoloniferous, upper parts erect. Leaves opposite, petiolate. Inflorescences umbel-like cymes, axillary or pseudoterminal; involucre globose to ovoid. Corolla zygomorphic, tubular to funnelform, white, pale yellow, or pink. Stamens two, with 2–3 staminodes; disc ring-like. Ovary superior, linear, 2-loculed, one locule fertile; stigma truncate or capitate. Capsule long-elliptic-lanceolate to linear (Wang et al. 1998).

The genus *Hemiboea* was established by C.B.Clarke in 1888, with *H. follicularis* C.B.Clarke designated as the type species. Taxonomic studies of *Hemiboea* have involved the description of new species and repeated taxonomic revisions. Molecular and morphological studies showed that *Metabriggsia ovalifolia* and *M. purpureotincta* are nested within *Hemiboea* and were formally transferred to the genus (Weber et al. 2011). In recent years, numerous new species within the genus have been discovered and described (e.g., Miao et al. 2022; Peng et al. 2022; Huang et al. 2024; Liang et al. 2025). Such continuous discovery of new species in these specific limestone habitats is not coincidental but intrinsically linked to explosive radiations, which were triggered by niche heterogeneity resulting from paleoclimatic fluctuations (Xu et al. 2021; Kong et al. 2022; Tan et al. 2026). This complex evolutionary history underpins the region’s extraordinary biodiversity and high endemism. Consequently, as of January 2026, according to the Gesneriaceae Resource Centre (GRC 2026), the genus is considered to comprise 47 species and five varieties.

In October 2014, during a field survey in Qichong National Nature Reserve, Hezhou, Guangxi, a distinctive population of *Hemiboea* was discovered in a dark, moist valley. This population is characterized by pubescence on its leaves and stems, a spherical involucre drying early before anthesis, a pale pink corolla, and a relatively late flowering and fruiting period. This unique combination of traits indicates that the population is clearly distinguishable from all other cogeneric species. Its status as a new species was validated by detailed morphological comparisons and molecular phylogenetic analyses, as elaborated below.

## Material and methods

### Morphological observation

This suspected new species was discovered during a field survey in Qichong National Nature Reserve in Hezhou, Guangxi. Its morphology and phenology were documented based on observations of ca. 30 mature individuals in the wild and 10 cultivated plants grown at the Guangxi Institute of Botany. Morphological comparisons with other species of *Hemiboea* were conducted using herbarium specimens and original literature. Physical specimens from the IBK herbaria and digitized type specimens from the Chinese Virtual Herbarium (https://www.cvh.ac.cn) and the JSTOR Global Plants database (plants.jstor.org), including specimens from K, E, P, MPU, and UC, as well as digital specimens from IBSC, PE, CDBI, IBK, GXMI, and KUN, were meticulously examined. Representative specimens examined for comparison include *H. subcapitata* (e.g., *A. Henry* s.n., GH00015880; *Y.L. Su & J.Q. Huang* 450331200822038LY, IBK00436371), *H. cavaleriei* (e.g., *P.J. Cavalerie* 492, E00135154; *W.B. Xu* s.n., IBK00407827), and *H. liana* (*B. Pan* PB2017092601, IBK00417133). Relevant literature on the closely related taxa, particularly *H. subcapitata*, *H. cavaleriei*, and *H. liana*, was also consulted (Li and Wang 2005; Wei et al. 2010; Huang et al. 2024). Voucher specimens of the putative new species have been deposited at the Herbarium of the Guangxi Institute of Botany (IBK).

### DNA extraction, sequencing, and assembly

Leaf samples for genomic sequencing were collected from the conservation nursery, including three individuals of this putative new species and one individual of each of the five known species (*H. liana*, *H. lutea*, *H. roseoalba*, *H. shimentaiensis*, and *H. subcapitata*). All samples were dried in silica gel immediately after collection. Genomic DNA was isolated using a modified CTAB technique (Doyle and Doyle 1987). Sequencing libraries with an insert size of approximately 350 bp were constructed and sequenced on the NovaSeq 6000 platform in paired-end 150 mode at Biomarker Technologies Corporation (Beijing, China). Each sample yielded more than 3.15 GB of raw data, which was filtered to remove adapters and low-quality reads using fastp v1.0.1 (Chen 2025).

*De novo* assembly of ribosomal DNA (ITS) and chloroplast genomes for all sequenced samples was conducted using GetOrganelle v1.7.7.1 (Jin et al. 2020) with the default settings. Complete ITS and chloroplast genome assemblies were obtained for the putative new species. For the remaining taxa, the complete ITS assemblies were generated for *H. lutea*, *H. roseoalba*, and *H. shimentaiensis*, whereas the chloroplast genome assemblies were generated for *H. liana*, *H. lutea*, *H. roseoalba*, *H. shimentaiensis*, and *H. subcapitata*. ITS regions were extracted from all ITS assemblies using ITSx 1.1.2 (Bengtsson-Palme et al. 2013). Chloroplast genomes were annotated using CPGAVAS2 (Shi et al. 2019), with *H. yongfuensis* (GenBank: NC_079573) as the reference. All newly generated ITS and complete chloroplast genome sequences have been deposited in the NCBI database (https://www.ncbi.nlm.nih.gov/nuccore).

### Phylogenetic analysis

To evaluate the monophyly and phylogenetic position of the putative new species, 33 published ITS sequences and 13 complete chloroplast genomes of *Hemiboea* were retrieved from NCBI and analyzed together with the newly generated data from this study. Species of *Lysionotus* and *Petrocosmea* were selected as outgroups, with *P. crinita* and *P. rotundifolia* used for chloroplast genome phylogeny, and *P. kerrii* and *P. nervosa* used for ITS phylogeny. GenBank accession numbers of all sequences used in the phylogenetic analyses are provided in Table 1.

**Table 1.** GenBank accession numbers of the ITS sequences and complete chloroplast genome sequences used in this study.

**Species**	**GenBank accession number**	
	**ITS**	**Complete chloroplast genome**
**Ingroup samples**		
*Hemiboea albiflora* X.G. Xiang, Z.Y. Guo & Zhao W. Wu	MN334629	/
*Hemiboea bicornuta* (Hayata) Ohwi	FJ501356	/
*Hemiboea cavaleriei* H. Lév.	FJ501355	/
*Hemiboea cavaleriei* var. *paucinervis* W. T. Wang & Z. Yu Li	MN334630	/
*Hemiboea crystallina* Y.M. Shui & W.H. Chen	MN334631	/
*Hemiboea fangii* Chun ex Z. Yu Li	HQ632979	NC_079575
*Hemiboea flaccida* Chun ex Z. Yu Li	JF697567	/
*Hemiboea follicularis* C.B. Clarke	HQ632982	/
*Hemiboea gracilis* Franch.	KY288038	/
*Hemiboea guangdongensis* (Z.Y. Li) X.Q. Li & X.G. Xiang	MF625025	/
***Hemiboea siccinvolucris***	**PV355626**,	**PX508911**,
	**PV355627**,	**PX508912**,
	**PV355628**	**PX508913**
*Hemiboea integra* C. Y. Wu ex H. W. Li	/	NC_079574
*Hemiboea kaiyangensis* T. Peng & S. Z. He	JN644335	/
*Hemiboea latisepala* H. W. Li	MN334636	/
*Hemiboea liana* Z.P. Huang, Y.B. Lu & B. Pan	MW035005	PX505290
*Hemiboea longgangensis* Z. Yu Li	HQ632986	/
*Hemiboea longzhouensis* W. T. Wang ex Z. Yu Li	HQ632985	/
*Hemiboea lutea* F. Wen, G. Y. Liang & Y. G. Wei	PV364908	PX505291
*Hemiboea magnibracteata* Y. G. Wei & H. Q. Wen	HQ632984	/
*Hemiboea malipoensis* Y. H. Tan	MN334639	NC_080279
*Hemiboea mollifolia* W. T. Wang	MN334640	/
*Hemiboea omeiensis* W. T. Wang	HQ632983	/
*Hemiboea ovalifolia* (W. T. Wang) A. Weber & Mich. Möller	HQ632980	NC_054358
*Hemiboea parvibracteata* W. T. Wang & Z. Yu Li	/	NC_080280
*Hemiboea pseudomagnibracteata* B. Pan & W. H. Wu	KY288035	/
*Hemiboea pterocaulis* (Z. Y. Li) J. Huang, X. G. Xiang & Q. Zhang	KY607397	NC_082106
*Hemiboea purpurea* Yan Liu & W. B. Xu	MN334644	NC_080277
*Hemiboea purpureotincta* (W.T.Wang) A.Weber & Mich.Moeller	HQ632981	NC_080281
*Hemiboea roseoalba* S. B. Zhou, Xin Hong & F. Wen	PV364909	PX505292
*Hemiboea rubribracteata* Z. Yu Li & Yan Liu	KY288039	/
*Hemiboea shimentaiensis* S. Y. Miao, Y. Q. Li & Tao Chen	PV364910	PX505293
*Hemiboea sinovietnamica* W. B. Xu & X. Y. Zhuang	PP647894	NC_079572
*Hemiboea strigosa* Chun ex W. T. Wang	MN334647	/
*Hemiboea subacaulis* Hand.-Mazz.	MN334658	NC_080278
*Hemiboea subacaulis* var. *jiangxiensis* Z. Yu Li	PV448327	PP816035
*Hemiboea subcapitata* C. B. Clarke	FJ501357	PX505294
*Hemiboea suiyangensis* Z. Yu Li, S. W. Li & X. G. Xiang	MN334659	NC_080282
*Hemiboea wangiana* Z. Yu Li	KY288046	/
*Hemiboea yongfuensis* Z. P. Huang & Y. B. Lu	MK441674	NC_079573
**Outroup samples**		
*Lysionotus chingii* Chun ex W. T. Wang	FJ501332	PQ468980
*Lysionotus petelotii* Pellegr.	HQ632974	PQ468970
*Petrocosmea kerrii* Craib	FJ501334	/
*Petrocosmea crinita* (W. T. Wang) Z. J. Qiu	/	PQ279903
*Petrocosmea nervosa* Craib	FJ501335	/
*Petrocosmea rotundifolia* M. Q. Han, H. Jiang & Yan Liu	/	PQ279902

DNA sequences were aligned in MAFFT 7.490 (Katoh and Standley 2013) using default parameters. Highly divergent regions in the alignment were identified and masked with the maskSegment function of AlignmentFilter 1.3.0 (Zhang et al. 2023), and sites with more than 50% gaps were removed using the degap function. The final ITS and complete chloroplast genome alignment matrices were assessed in MEGA 11 (Tamura et al. 2021) to calculate the consistency index (CI), retention index (RI), and homoplasy index (HI). Phylogenetic incongruence between the ITS and complete chloroplast genome datasets was assessed using the Incongruence Length Difference (ILD) test in PAUP v4.0a169 (Swofford 2002) with 1,000 permutation replicates. Phylogenetic trees were constructed using both maximum likelihood (ML) and Bayesian inference (BI) approaches. Optimal substitution models were selected in MrModeltest 2.3 (Nylander 2004) based on the Akaike Information Criterion (AIC; Akaike 1974). The ML analyses were conducted in RAxML-HPC (Stamatakis 2014), whereas the BI analyses were performed in MrBayes v3.2.6 (Ronquist et al. 2012) using four Markov chain Monte Carlo (MCMC) chains run for 10,000,000 generations, with sampling every 100 generations.

## Results

### Phylogenetic analyses

**Figure 1.**
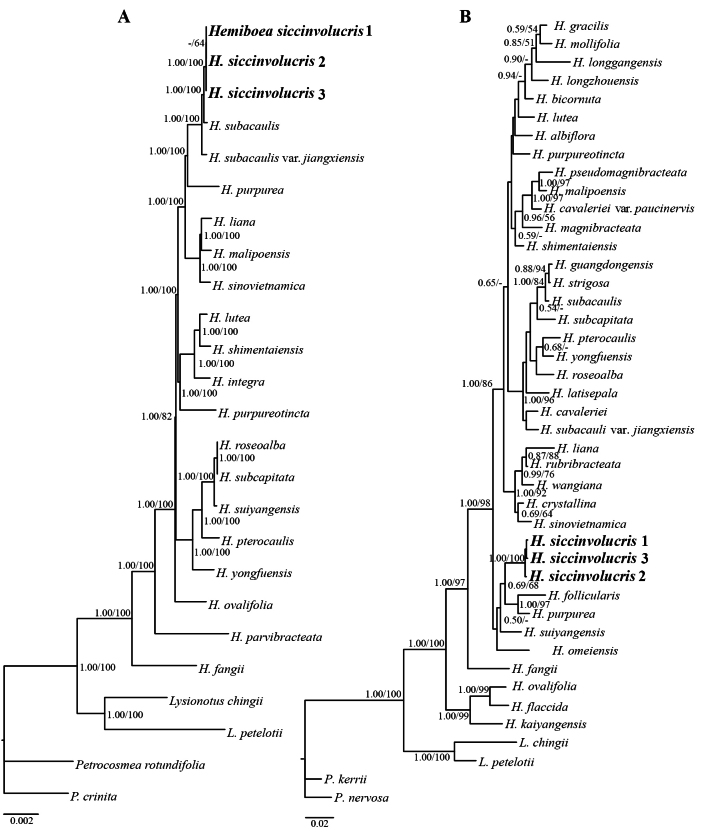
Bayesian inference phylogenetic trees of *Hemiboea* based on **A**. Complete chloroplast genome sequences (21 ingroup samples); **B**. Nuclear ITS sequences (39 ingroup samples), with four outgroup taxa included in each analysis. Nodal support is indicated as posterior probabilities (≥ 0.50) and bootstrap values (≥ 50%); a dash (–) denotes support values below 50%. The newly described species *H. siccinvolucris* is highlighted in bold.

The aligned ITS and complete chloroplast genome matrices contained 640 and 153,682 characters, respectively, including 233 and 7,038 variable sites and 154 and 2,726 parsimony-informative sites. The CI, RI, and HI values were 0.615, 0.715, and 0.385 for the ITS dataset and 0.886, 0.870, and 0.114 for the whole chloroplast genome dataset. The ILD test indicated significant incongruence between the ITS and entire chloroplast genome matrices (*p* = 0.001, indicating strong conflict between the two datasets), implying their unsuitability for concatenated analysis. Phylogenetic analyses were conducted for both datasets using ML and BI under the best-fit nucleotide substitution models (GTR+I+G for ITS and GTR+I+R for the chloroplast genome data) (Fig. 1).

The ML and BI analyses produced congruent topologies and supported the monophyly of *Hemiboea* (bootstrap [BS] = 100%; posterior probability [PP] = 1.00), within which all sampled individuals of the putative new species (i.e., *H. siccinvolucris*) formed a lineage (BS = 100%; PP = 1.00). In the chloroplast genome phylogeny, *H. siccinvolucris* was identified as a sister group to *H. subacaulis*, with strong support (BS = 100%; PP = 1.00), and they together formed a strongly supported clade with *H. subacaulis* var. *jiangxiensis* (BS = 100%; PP = 1.00) (Fig. 1A). In the ITS phylogeny, all sampled individuals of *H. siccinvolucris* formed a lineage that was sister to the clade of *H. follicularis* and *H. purpurea*, with moderate support (BS = 68%; PP = 0.69) (Fig. 1B). Phylogenetic analyses based on both ITS and chloroplast genomes consistently recovered *H. siccinvolucris* as a well-supported lineage, coinciding with the presumption that it represents a new species.

### Taxonomic treatment

#### Hemiboea siccinvolucris

Taxon classificationPlantaeLamialesGesneriaceae

B.Pan & Y.B.Lu sp. nov.

928D6A28-C607-52CB-A3FA-EA1189C3FB0A

urn:lsid:ipni.org:names:77379931-1

Figs 2, 3

##### Chinese name.

Gān bāo bàn shuò jù tái (干苞半蒴苣苔).

##### Diagnosis.

This new species is morphologically similar to *H. cavaleriei*, *H. subcapitata*, and *H. liana* but can be distinguished by a set of vegetative and reproductive traits. It is 30–55 cm tall, with pubescent stems (vs. 20–150 cm tall, glabrous in *H. cavaleriei*; 10–40 cm tall, glabrous to pubescent in *H. subcapitata*; 10–46 cm tall, glabrous in *H. liana*). Leaf differences are densely pubescent adaxially and sparsely pubescent abaxially (vs. adaxially glabrous to sparsely pubescent and abaxially glabrous in *H. cavaleriei*; adaxially nearly glabrous to pubescent and abaxially glabrous to pubescent in *H. subcapitata*; glabrous on both surfaces in *H. liana*). It bears terminal 1–3-flowered cymes, with involucres drying early before anthesis and occasionally persisting at the corolla base (vs. pseudoterminal 3–12-flowered cymes in *H. cavaleriei*; pseudoterminal or axillary, 3–10- or more flowered cymes in *H. subcapitata*; terminal, usually 1-flowered cymes in *H. liana*, with none of these species recorded with early involucre desiccation). It flowers from November to December (vs. August–October in *H. cavaleriei* and *H. subcapitata*; November–January of the following year in *H. liana*). More diagnostic characters are comparatively shown in Table 2.

**Figure 2.**
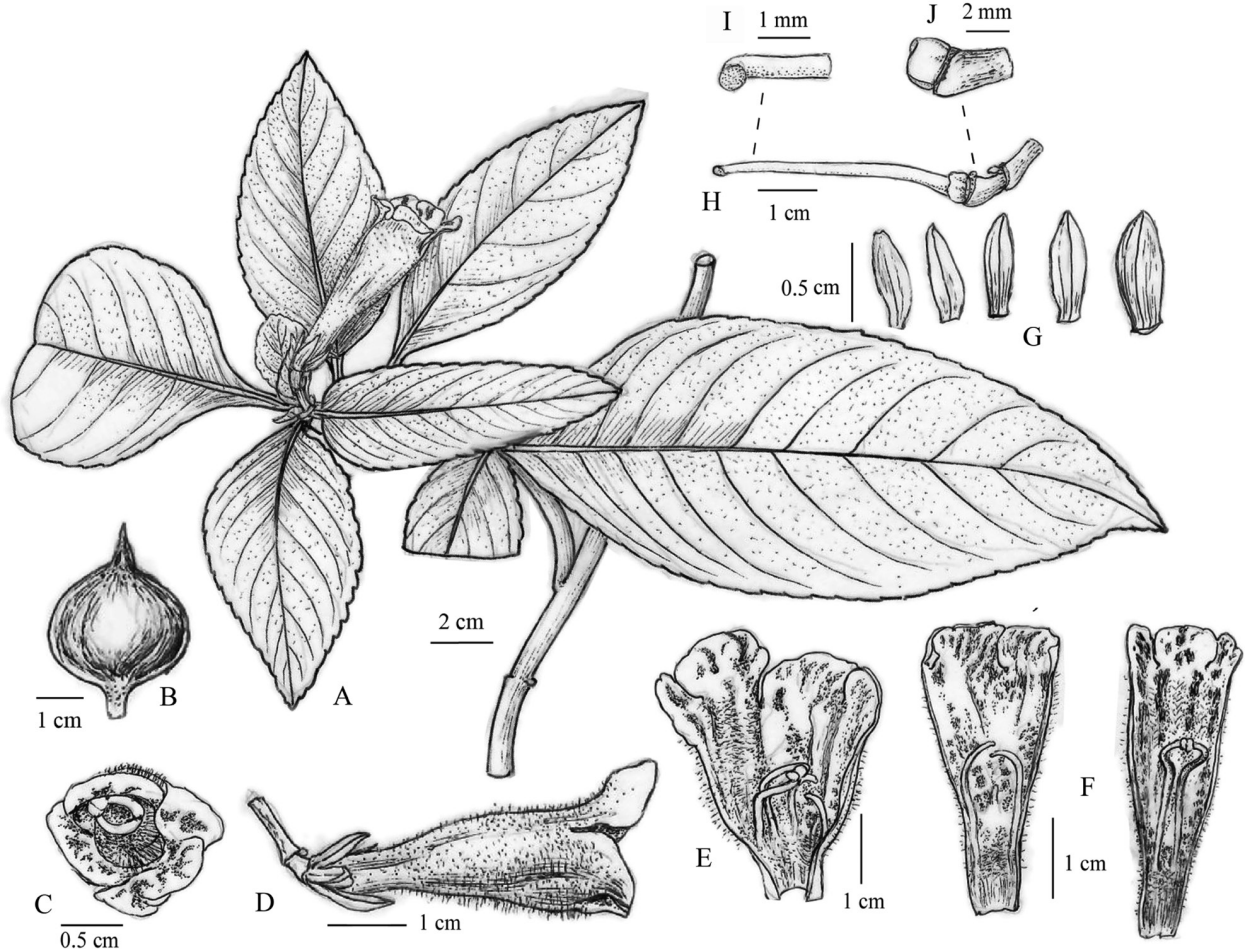
*Hemiboea siccinvolucris* B.Pan & Y.B.Lu, sp. nov. **A**. Habit; **B**. Involucre; **C**. Front view of flower; **D**. Side view of flower; **E**, **F**. Opened corolla with stamens and staminodes; **G**. Calyx; **H**. Pistil; **I**. Upper style and stigma; **J**. Disc.

**Figure 3.**
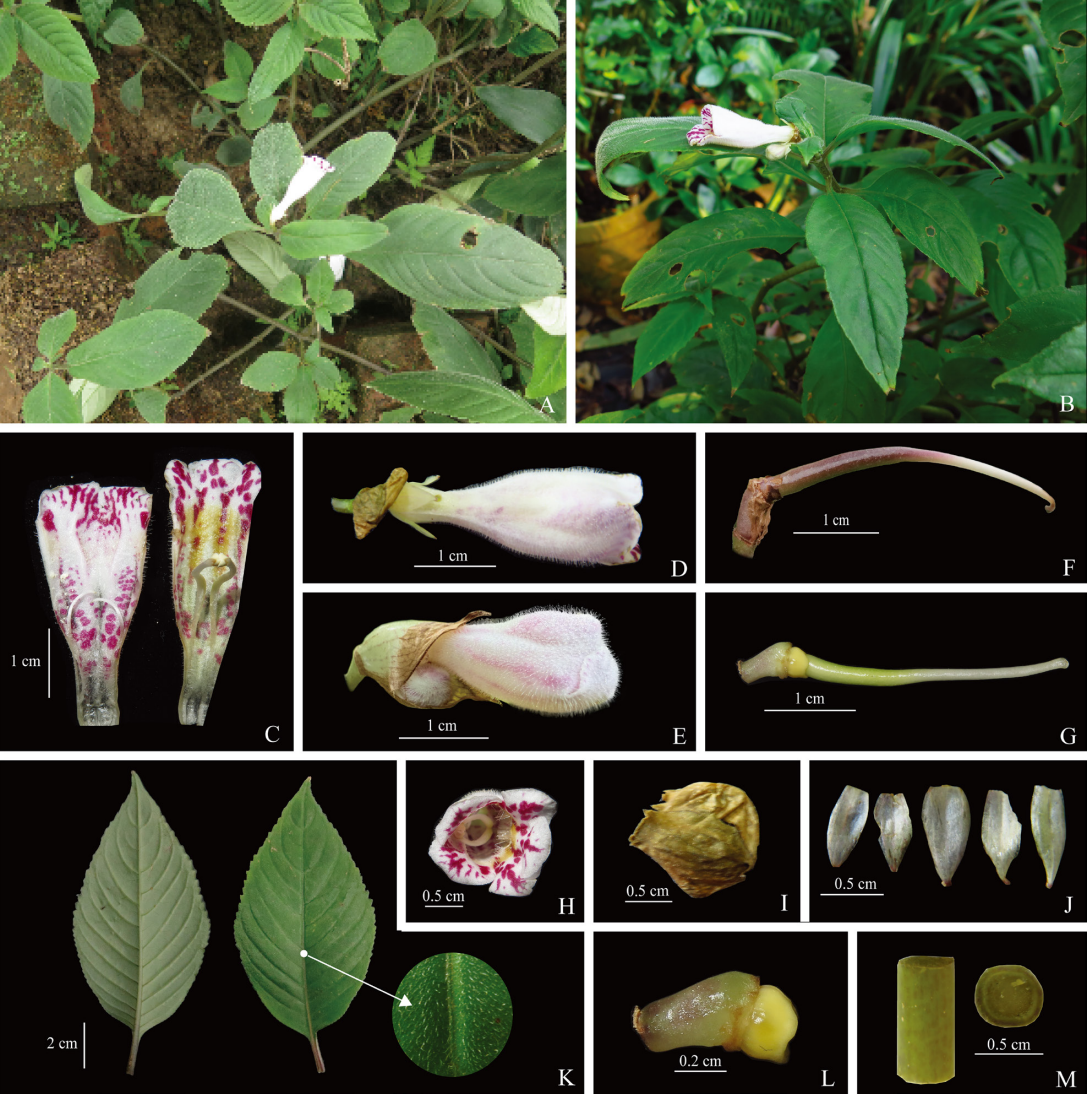
*Hemiboea siccinvolucris* B.Pan & Y.B.Lu, sp. nov. **A**. Habit; **B**. Flowering individual; **C**. Opened corolla with stamens and staminodes; **D, E**. Side view of flower; **F**. Capsule; **G**. Pistil with disc; **H**. Front view of flower; **I**. Dried involucre; **J**. Opened calyx; **K**. Leaf blade; **L**. Disc; **M**. Cross-section of the stem.

**Table 2.** Morphological comparisons of *Hemiboea siccinvolucris* and the similar species *H. cavaleriei*, *H. subcapitata*, and *H. liana*.

Characters	*H. siccinvolucris*	*H. cavaleriei*	*H. subcapitata*	*H. liana*
**Stems**	30–55 cm, pubescent	20–150 cm, glabrous	10–40 cm, glabrous to pubescent	10–46 cm, glabrous
**Leaf**	green, densely pubescent on the adaxially, sparsely pubescent on the abaxially	adaxially green, abaxially pale green or tinged with purple, adaxially glabrous to sparsely pubescent, abaxially glabrous	adaxially green, abaxially pale green or tinged with purple, adaxially nearly glabrous to pubescent, abaxially glabrous to pubescent	adaxially green, abaxially lilac, glabrous on both sides
**Petiole**	1.3–3.2 cm, pubescent	0.5–6.5 cm, glabrous	1–7 (–9) cm, glabrous	2–2.5 cm, glabrous
**Lateral veins**	9–11 on each side of midrib	6–14 on each side of midrib	5–7 on each side of midrib	5–8 on each side of midrib
**Cyme**	terminal, with 1–3 flowers	pseudoterminal, 3–12 flowered	pseudoterminal or axillary, 3–10 or more flowered	terminal, usually one flowered, occasionally two flowered
**Involucre**	spheroidal, drying early before anthesis, occasionally persisting at corolla base	spheroidal, early involucre desiccation not recorded	spheroidal, early involucre desiccation not recorded	trigonous, early involucre desiccation not recorded
**Corolla**	pale pink outside, purple-spotted inside	pale yellow to white outside, purple-spotted inside	white outside, purple-spotted inside	pink-spotted outside, white, with scattered pink and purple spots inside
**Anthers**	elliptic,1.5–1.7 mm long	elliptic, 3–3.2 mm long	long-elliptic, 3.5–4.5 mm long	semicircular, 2–2.5 mm long
**Stigma**	reniform	obtuse	obtuse	shallowly bilobed
**Flowering**	November to December	August to October	August to October	November to January the following year

Note: Morphological characters for the compared species were retrieved from the following sources: *H. cavaleriei* and *H. subcapitata* (Wang et al. 1998), and *H. liana* (Huang et al. 2024).

##### Type.

China • Guangxi Zhuang Autonomous Region: Hezhou City, Zhaoping County, Qichong Nature Reserve, 24°16'06.84"N, 110°49'50.97"E, elev. ca. 171 m, growing on limestone hillsides, 5 Nov. 2014, *B. Pan et al. QC20141105022* (holo-type: IBK00470681; isotypes: IBK00470682, IBK00470683).

##### Description.

Perennial herb. Stems 30–55 cm tall, subterete, green and sparsely purple-spotted, pubescent, simple, with 5–12 nodes. Leaves opposite, 6–8 per stem, leaf blade chartaceous, elliptic or ovate-elliptic, green, densely pubescent adaxially, sparsely pubescent abaxially, 5.2–12.5 × 3.2–6.1 cm, margin shallowly undulate or crenate, apex acuminate, base cuneate or narrowly cuneate, often asymmetrical; petiole 1.3–3.2 cm long, approximately 2–3 mm in diameter, semi-terete, pubescent; veins slightly sunken adaxially, raised abaxially, lateral veins 9–11 on each side of midrib. Cyme terminal, with 1–3 flowers; peduncle 1–1.3 cm long, glabrous, sparsely purple-spotted; involucre spheroidal, drying early before anthesis. Pedicel 2–4 mm long, glabrous. Calyx white, 5-parted from the base, segments ovate or linear-lanceolate, 5–6 × 2–3 mm, glabrous, membranous when dry. Corolla pale pink, outside densely glandular-puberulent and with scattered pink spots, inside pink throughout with prominent purple striations and maculations, and ring of hairs 4.5–6.0 mm above base; corolla tube 3.6–4.2 cm long, 1.3–1.5 cm in diameter at the mouth, 4–5 mm in diameter at the base, the upper sector of the adaxially lip is pinkish with scattered purple spots; the lower sector connecting to the abaxial lip is pale yellow, densely pubescent with ca. 2 mm white trichomes, and sparsely dotted with purple spots; limb distinctly 2-lipped, adaxially lip 2-lobed, lobes semirotund, 5–6 mm long; abaxially lip 3-lobed, lobes oblong, 5–7 mm long. Stamens 2, filaments linear, glabrous, geniculate from the middle, adnate to 1.2–1.4 cm above corolla base, 1.5–1.6 cm long; anthers elliptic, 1.5–1.7 mm long, coherent at apex; staminodes 3, lateral ones 6–7 mm long, the middle one 1–2 mm long. Disc ringlike, 2–2.2 mm high. Pistil 1.9–2.7 cm long, ovary linear, 7–12 mm long, 1.5–1.8 mm in diameter, glabrous; style 1.2–1.5 cm, stigma reniform. Capsule linear-lanceolate, ca. 2 cm long, base width 3–4 mm, acuminate at apex, slightly curved, glabrous.

##### Phenology.

Flowering occurs from November to December; fruiting occurs from November to January of the following year.

##### Etymology.

The specific epithet refers to the characteristic involucre, which becomes dry and scarious before the flowers open.

##### Distribution and habitat.

*H. siccinvolucris* is currently only known from low-altitude, moist gully margins in Qichong Nature Reserve, Zhaoping County, Hezhou City, Guangxi Zhuang Autonomous Region, South China. It grows in association with species including *Illicium simonsii* Maxim., *Impatiens tubulosa* Hemsley, *Ophiorrhiza pumila* Champ. & Benth., *Elatostema acuminatum* (Poir.) Brongn., *Alsophila spinulosa* (Wall. ex Hook.) R.M.Tryon, and *Dicranopteris pedata* (Houtt.) Nakaike.

##### IUCN red list category.

*H. siccinvolucris* is currently known solely from its type locality, with fewer than 200 individuals recorded. However, it cannot be excluded that there are undiscovered populations in similar habitats elsewhere. Due to limited field surveys, key parameters such as the population size, reproductive capacity, and habitat adaptability of *H. siccinvolucris* have not been quantitatively assessed. According to the IUCN Red List Categories and Criteria (IUCN 2024), this newly described species should be provisionally classified as Data Deficient (DD).

## Discussion

As detailed in the Diagnosis section, *H. siccinvolucris* is readily distinguishable from its close relatives (*H. cavaleriei*, *H. subcapitata*, and *H. liana*) by several key morphological differences. The newly described species *H. siccinvolucris* is distinguished by pubescent leaves and early-drying involucres. In *Hemiboea*, leaf pubescence is a stable morphological trait, either present or absent. Several other *Hemiboea* species, such as *H. glandulosa*, *H. fangii*, *H. gracilis*, and *H. omeiensis*, also possess pubescent adaxial leaf surfaces, but *H. siccinvolucris* can be distinguished by its combination of dense adaxial pubescence and sparse abaxial pubescence, along with other characters (Li and Wang 2005; Wei et al. 2010). Additionally, early involucre desiccation is rare in *Hemiboea*, observed only in a few congeneric species, such as *H. kaiyangensis*, which exhibits early involucre abscission. The combination of these distinct traits and other morphological features, including stems, petioles, lateral veins, corollas, anthers, and stigmas, allows for clear and reliable differentiation of this new species from other known ones.

Phylogenetic analyses were conducted using the ITS sequences from 33 *Hemiboea* species and complete chloroplast genome sequences from 13 species. Both nuclear and plastid datasets recovered *H. siccinvolucris* as a well-supported lineage. In the chloroplast phylogeny, it is sister to *H. subacaulis*, whereas in the nuclear ITS tree, it forms a distinct clade sister to the combined clade of *H. follicularis* and *H. purpurea*. Based on these phylogenies, *H. siccinvolucris* is closely related to *H. subacaulis*, *H. follicularis*, and *H. purpurea*, but it is clearly distinct morphologically: stem 30–55 cm tall (vs. 5–22.5 cm in *H. subacaulis*); calyx deeply divided to the base into free, long-elliptic or linear-lanceolate lobes (vs. fused above the middle in *H. purpurea* and *H. follicularis*); corolla pale pink with purple stripes (vs. uniformly purple corolla in *H. purpurea* and white corolla in *H. follicularis*); corolla tube densely glandular-pubescent externally (vs. glabrous in *H. follicularis* and *H. purpurea*); leaves pubescent on both surfaces (vs. glabrous in *H. follicularis* and *H. purpurea*). Phylogenetic analyses and distinct morphological traits confirm *H. siccinvolucris* as a clearly independent new species within *Hemiboea*. In addition, the nuclear and plastid phylogenetic trees show some incongruence. The chloroplast genome matrix has higher consistency (CI: 0.886 vs. 0.615) and lower homoplasy (HI: 0.114 vs. 0.385) than the nuclear ITS matrix, indicating a more reliable phylogenetic signal. Nuclear-plastid discordance is common in Gesneriaceae. For example, in the subtribe Didymocarpinae, Yang reported that ancient hybridization and gene flow contribute to topological discordance between nuclear and plastid genomes (Yang et al. 2023a). Similarly, in *Henckelia*, cytonuclear discordance has been attributed to inadequate lineage sorting and hybridization, often associated with polyploidization (Yang et al. 2023b). These examples suggest that the evolutionary history of *H. siccinvolucris* may also involve reticulate processes such as hybridization or introgression. At the same time, the inconsistent phylogenetic placement of *H. siccinvolucris* between the ITS and chloroplast trees may also reflect methodological limitations, such as a limited number of informative sites, restricted taxon sampling, or other analytical factors. Further studies incorporating broader taxon sampling and higher-resolution molecular markers are needed to clarify the precise phylogenetic placement of *H. siccinvolucris*.

## Supplementary Material

XML Treatment for Hemiboea siccinvolucris

## Additional information

### Artificial Intelligence (AI) use

The authors accept full responsibility for the content of the manuscript, including the disclosure of any use of AI.

No AI tools were used in the preparation of this manuscript.

### Author ORCIDs

Y. Fang https://orcid.org/0009-0001-3637-0762

B. Pan https://orcid.org/0000-0003-0996-524X

P.-W. Li https://orcid.org/0000-0002-8195-1478

X.-M. Qin https://orcid.org/0000-0002-9977-254X

Q. Zhang https://orcid.org/0000-0002-2217-2407

Y.-B. Lu https://orcid.org/0000-0002-8635-3354

### Data availability

All of the data that support the findings of this study are available in the main text.
